# Healthy lifestyle behaviors and gynecological cancer awareness in women academicians: a descriptive and correlational study

**DOI:** 10.1007/s00404-024-07907-2

**Published:** 2025-03-15

**Authors:** Rabiye Akin Işik, Ayşe Arikan Dönmez, Füsun Terzioğlu

**Affiliations:** 1 Faculty of Nursing, Hacettepe University, Ankara, Turkey; 2 Faculty of Nursing, Hacettepe University , Ankara, Turkey; 3 Independent Researcher, Ankara, Turkey

**Keywords:** Women, Academician, Healthy life behaviors, Gynecological cancer awareness

## Abstract

**Objective:**

To determine the healthy lifestyle behaviors (HLBs) and gynecological cancer awareness (GCA) levels of women academicians and to investigate the correlation between HLBs and GCA.

**Methods:**

A descriptive correlational study design was used to determine the healthy life behaviors and gynecological cancer awareness levels of women academicians and to investigate the correlation between them. A total of 353 women academicians were included between 1st March 2020 and 1st January 2021. The data were collected using Introductory Form, Health Promoting Lifestyle Profile II, and Gynecological Cancer Awareness Scale.

**Results:**

The women’s healthy life behaviors levels were close to moderate and gynecological cancer awareness levels were high. The median healthy life behaviors score was significantly higher in women who worked as an assistant professor, considered the age of menarche and menopause as risk factors for women cancers, consumed regular snacks, received information about GCs, and had regular pap-smears (*p* < .05). The median gynecological cancer awareness score was significantly higher in women who worked as an assistant professor, considered the age of menarche and menopause as risk factors for women cancers, received information about GCs, and experienced pregnancy process (*p* < .05). In addition, as women’s healthy life behaviors levels increased, their gynecological cancer awareness levels also increased. The healthy life behaviors score was positively and moderately associated with the gynecological cancer awareness score (*p* < .05).

**Discussion:**

Our findings highlight the potential to promote monitoring of women’s healthy lifestyle behaviors and gynecological cancer awareness in the community by planning effective interventions.

## What does this study add to the clinical work

**Table:** 

This study highlights that HLB and GCA levels are associated with working position, exercising regularly, consuming regular meals and snacks, regular gynecological examination, and pap-smear, getting information about GCs and knowing risk factors.	
Raising awareness of all women in the society about early diagnosis methods, symptoms and risk factors, routine control for GC may be contributed to adopt HLB in their lives.	

## Introduction

Current global cancer data show that the cancer burden is increasing every year. The International Agency for Research on Cancer (IARC) reported that an estimated 19.3 million new cancer cases and almost 10 million cancer deaths occurred worldwide by 2020 [1]. Gynecological cancers (GCs), one of the cancers types whose number of cases increase rapidly every year, are of critical importance for women’s health [2]. According to the 2020 IARC data, among all cancer types in women, the 1st, 4th, 5th, and 7th rank of women cancers are: breast; cervix uteri, corpus uteri and ovary [3]. In the last 1 year, among all cancer types in women in Turkey, the breast ranked 1st, cervix uteri 2nd, corpus uteri 7th, ovary 9th [4].

Each type of GC has its own risk factors and having healthy lifestyle behaviors (HLBs) can be effective in reducing these risks [5]. While factors such as early onset of menstruation, delayed menopause, infertility and obesity are important risk factors for endometrial cancer [6], factors such as human papilloma virus (HPV), sexual intercourse at an early age or with multiple partners, smoking, and multiple births are known to be associated with cervical cancer [7]. Advanced age, being overweight or obese, having children later or never having a full-term pregnancy, receiving hormone therapy after menopause, having a family history of ovarian, breast or colorectal cancer are the main known risk factors for ovarian cancer [8]. Although the exact cause of vulvar and vaginal cancers is not clearly known, similar factors such as advanced age, HPV exposure, smoking, presence of precancerous lesions or disease in the vulva increase the risk as in other GCs [5]. The World Health Organization reports that about one-third of cancer deaths are due to modifiable lifestyle behaviors such as tobacco use, high body mass index (BMI), alcohol consumption, low fruit and vegetable intake, and lack of physical activity [2]. In general, most of the afore-mentioned risk factors for each type of GC can be modified. In other words, these cancers can be prevented or diagnosed at an early stage by adopting HLBs and integrating them into life. For example, HLBs such as regular exercise and healthy nutrition, vaccination for HPV, regular pap-smear tests, smoking cessation, regular medical check-ups, safe sexual intercourse practices, regular gynecological examinations are critical to reduce the risk of developing GC and to ensure early detection of the disease [9].

Primary (individual healthy lifestyle behaviors, physical activity) and secondary preventive health services (early diagnosis and screening) are recommended to reduce cancer incidence and mortality. One of the most important factors to prevent GCs is to have gynecological cancer awareness (GCA) [10]. GCA means that the society and individuals have knowledge about GCs, symptoms, risk factors, prevention methods and the importance of early diagnosis and awareness of these issues [1, 2]. The GCA enables women to recognize their own body, to know normal and abnormal changes in their reproductive organs, to recognize abnormalities quickly and to seek appropriate and timely health care services [11]. This awareness encourages women to undergo regular screening tests (e.g., Pap smears and HPV tests) and thus increases the likelihood of detecting cancer at an early stage. It also encompasses knowledge about adopting healthy lifestyles and controlling risk factors (e.g. smoking, obesity) [12, 13]. By fostering GCA, women can enhance the probability of early diagnosis by recognizing symptoms and seeking medical attention when necessary. This, in turn, elevates the efficacy of treatment and disease survival rates [12, 14, 15]. Studies on GCs show that women do not know the symptoms of cervical [16] and ovarian cancer [17], and risk factors associated with endometrial cancer [18], and therefore they have low GCA [19].

Healthy lifestyle behaviors (HLBs) refer to the attitudes and behaviors that individuals integrate into their lifestyles to protect and improve their health and prevent diseases [20]. Adequate and balanced nutrition, regular physical activity, stress management, avoidance of smoking and alcohol use, adequate sleep, personal hygiene, environmental cleanliness, routine health checks and positive social relationships form the basis of these behaviors [21, 22]. These behaviors are aimed at supporting the general well-being of the individual in the areas of physical, mental and social health. Moreover, HLBs have an important place in the prevention of cancer, which is the leading cause of death in developed and developing countries [20, 23]. In addition, it is reported that women’s HLBs influence the development of GCs [22, 24]. Prevention of GCs includes some measures such as avoiding risky behaviors, smoking and alcohol, gaining HLBs such as a balanced diet, exercising regularly, and avoiding environmental risk factors [10, 22, 25, 26].

There is no study examining the relationship between GCA and HLB among women academicians in Turkey. However, results from the studies on the general population of women in the community, have reported that women’s GCA is generally not at the desired level, even though there are GC screening programs in Turkey that are free of charge and covered by the health insurance system [12, 14, 22, 27–30]. Atlas and Er Güneri (2022) [31] found that 85.8% of women consulted a doctor only when they had a problem with their reproductive organs and 16.3% did not go to GC screenings because of the fear they experienced on the delivery table. Teskereci et al. (2021) [14] reported that women who did not have gynecological examinations and Pap smear tests and who did not have information about GC had lower GCA than others. In Öztürk et al. (2021) [13] study, it was pointed out that women with higher education level and income, having gynecological complaints, fearing cancer, undergoing gynecological examination (including Pap test), performing vaginal self-examination and needing information about cancer had higher awareness and knowledge about gynecological cancer. Remarkably, they found that even among women with a high level of GCA, there was a lack of knowledge, such as not having regular examinations, not identifying symptoms and being unaware of the risk factors for these cancers. In a pre-test-post-test controlled study examining the effect of education about cervical cancer and HPV using the PRECEDE education model on women’s HLBs and beliefs, it was reported that at the end of the training, HLB, health motivation of women in the study group significantly increased compared to the control group, while their perception of barriers to Pap-Smear test decreased. It was also found that the rate of Pap-Smear testing increased among women in the study group due to increased knowledge and awareness.

Considering the increase in cancer incidence and prevalence, GCs will undoubtedly continue to be an important global health problem [32]. In addition to early diagnosis and screening in GCs, it is crucial to raise awareness among women about its causes, risk factors and symptoms, and to create behavior change. Accordingly, academicians, who are responsible for conducting education and research activities and providing public services at universities, play a key role in creating HLBs in society [33]. Exploring the GCA of women academicians and determining their HLBs is of great importance in terms of better understanding of these cancers, strengthening research processes and creating a more effective awareness in the society. The awareness of women academicians in this field and their current HLBs have an important role in the development of early diagnosis and prevention strategies. The fact that women academicians have high GCA and have adopted HLBs ensures the dissemination of correct information in the society. They can correct misconceptions about GCs and support the dissemination of protective and/or preventive HLBs. They exchange information with a large part of the population by interacting them and are also critical role models for young people and their students. On the other hand, women academicians are accepted as role models in protecting women’s social rights, leading a healthy life and taking responsibility in this regard [34]. Since they represent a high level of education group and interact with many women in society, including university students, they are expected to have acquired HLBs and be aware of cancer. Determining the level of GCA and HLBs for cancer prevention or protection also contributes to identifying the knowledge gaps of women academicians and the areas that need to be developed. In this way, the training needs of women academicians can be determined and their potential to contribute to scientific studies can be increased by providing the necessary training or resources. From this point of view, this study aimed to determine the HLBs and GCA levels of women academicians, and to investigate the correlation between HLBs and GCA.

### Research questions

1. What are the HLBs and GCA levels in women academicians?
2. Do HLBs and GCA levels differ by socio-demographic characteristics?
3. Is there a correlation between HLBs and GCAs scores in women academicians?

## Methods

### Study design

A descriptive and correlational design was used to examine the HLBs and GCA of women academicians.

### Study sample

The population of the study consisted of 10,000 women academicians working at state and foundation universities in Ankara. The study participants were recruited using convenience sampling. The participants were included if they (a) worked as a lecturer or faculty member at state and foundation universities in Ankara (b) were able to speak and read Turkish, and (c) had volunteered to participate in the study. The sample of the study was calculated as 264 people with 90% confidence level and 5% margin of error, according to the known population sampling method. The minimum sample size was calculated as 264 participants with 90% confidence level and 5% margin of error, according to the known population sampling method [35].

A total of 370 eligible women academicians were reached between 1st March 2020 and 1st January 2021. However, 17 of them were excluded because they refused to participate (*n* = 9) and filled in the data collection forms incompletely (*n* = 8). Therefore, 353 women academicians were included in the study.

### Outcome measures

#### Descriptive characteristics form

The form was developed by researchers based on the previous studies [36–38]. This form consisted of 33 questions about the participants’ descriptive characteristics (age, height, weight, BMI, university, and faculty), HLBs (smoking and alcohol consumption, exercising, and nutrition) and GCs.

#### Health promoting lifestyle profile II

The scale was developed by Walker and Hill-Polerecky (1996) [39] to determine health promoting lifestyle. It comprises 52 items classified into six dimensions: (1) spiritual development, (2) health responsibility, (3) physical activity, (4) nutrition, (5) interpersonal relationships and (6) stress management. Each item is measured on a Likert-type scale ranging from 1 (never) to 4 (routinely). This means that the total scores range from 52 to 208. Higher scores indicate a higher level of health promoting lifestyle. The Turkish validity-reliability study of the scale was performed by Bahar et al. (2008) [40]. The Turkish version of the scale has good psychometric properties with good reliability and validity; Cronbach’s alpha for the whole scale is 0.94, ranging from 0.64 to 0.80 for each dimension. In the present study, the total scale had a Cronbach’s alpha of 0.92, ranging from 0.685 to 0.876 for each dimension.

#### Gynecological cancer awareness scale

The scale was developed by Alp Dal and Ertem (2017) [10] to measure GCA levels in women. It comprises 41 items classified into four dimensions: (1) Routine control and serious disease perception awareness in GCs (2) GC risk awareness (3) Awareness of prevention from GCs (4) Early diagnosis and information awareness in GCs. Each item is measured on a Likert-type scale ranging from 1 (strongly disagree) to 5 (strongly agree). This means that the total scores range from 41 to 205. Higher scores indicate a higher level of awareness. The scale has good psychometric properties with good reliability and validity; Cronbach’s alpha for the whole scale is 0.94, ranging from 0.70 to 0.97 for each dimension. In the present study, the total scale had a Cronbach’s alpha of 0.91, ranging from 0.623 to 0.927 for each dimension.

### Data collection procedure

Data were collected by online survey method via Google Forms. The first part of the online survey included information about the study. The participants were given access to the data collection forms after reading the information about the study and marking the field declaring that they agreed to participate in the study. After the participants completed the survey, they clicked the submit button and the answers were collected in the common e-mail account used by the researchers.

### Ethical considerations

This study was approved by the Non-Interventional Clinical Trials Ethics Committee of Hacettepe University (approval number: GO19/1059). In addition, official institution permission was obtained from universities in Ankara. To ensure anonymity, no other identifying information that could have revealed names or identities was collected. All data were kept secure and confidential and was accessed by the research team only.

### Data analysis

Data were analyzed using the SPSS version 23 (SPSS Inc, Chicago, Illinois). The normality distribution was examined with Kolmogorov–Smirnov test, histogram, and normal Q-Q plots. Since the normality test revealed that the variables did not conform to the normal distribution, non-parametric tests (Mann–Whitney U and Kruskal–Wallis and Spearman Correlation tests with median values) were used in the analyses. The descriptive statistics (frequency, means, and standard deviations) were calculated for demographic characteristics and the mean scores for the whole and sub-dimensions of the scales. Mann–Whitney U and Kruskal–Wallis tests were used for comparisons between groups. The Spearman correlation test was used to evaluate the relationship between continuous measurements. Statistical significance level was accepted as *p* < 0.05 at 95% confidence interval.

## Results

### Descriptive characteristics of the participants

The mean age of the participants was 36.54 (SD = 10.03) years and BMI average was (SD = 3.99). The majority of the participants did not smoke (85.8%) or drink alcohol (63.2%). While 61.2% of the participants did not exercise regularly, 51% reported that they did not regularly consume snacks. A considerable majority of the participants did not consider both the age of menarche (53%) and having a family history of women cancer (71.4%) to be a risk factor for GCs. In addition, 56.7% stated that they did not have a regular gynecological examination and 60.3% did not have a pap-smear test. The considerable majority stated that they did not regular exercise (61.2%), consume regular main meal (51%) and snack meal (69.4%) (Tables 1 and 2).

**Table 1:** Socio-demographic characteristics of female academics (*n* = 353)

Variables	Mean ± SD	
	x̄ ± ss	
Age	36.54 ± 10.03	
Size	164.45 ± 5.84	
Weight	62.72 ± 11.04	
BMI	23.16 ± 3.99	

Title	n	%
Research assistant	130	36.8
Research assistant PhD	19	5.4
Lecturer	52	14.7
Assistant professor	77	21.8
Associate professor	30	8.5
Professor	45	12.7
Smoking (active smoking)		
Yes	50	14.2
No	303	35.8
Alcohol (current alcohol intake)		
Yes	130	36.8
No	223	63.2
Chronic disease		
Yes	81	22.9
No	272	77.1
Regular exercises		
Yes	137	38.8
No	216	61.2
Regular main meal consumption		
Yes	173	49
No	180	51
Regular snack meal consumption		
Yes	108	30.6
No	245	69.4
Women’s cancer in the family		
Yes	59	16.7
No	294	83.3

**Table 2:** Characteristics of women related to gynecologic cancers

Age		
Yes	245	69.4
No	108	30.6
Menarche’ age		
Yes	166	47
No	187	53
Menapouse’ age		
Yes	263	74.5
No	90	25.5
Family history of gynecologıcal cancer		
Yes	101	28,6
No	252	71.4
Regular gynecological examination		
Yes	153	43.3
No	200	56.7
Regular pap-smear examination		
Yes	140	39.7
No	213	60.3
Risk diagnosis of gynecological cancers		
Yes	38	10.8
No	315	89.2
Information on gynecological cancers		
Yes	209	59.2
No	144	40.8
Menstruation status		
Regular	279	79.0
Irregular	28	7.9
Menapouse		
Yes	44	12.5
No	309	87.5
Diagnosing gynecological cancer		
Yes	7	2
No	346	98
Polygamy		
Yes	8	2.3
No	345	97.7
Having polygamy in the past		
Yes	46	13
No	307	87
Pregnancy experiencing		
Yes	183	51.8
No	170	48.2
Infertility diagnosis		
Yes	21	5.9
No	332	94.1
Using COCs		
Evet	167	47.3
Hayır	186	52.7
STI diagnosis		
Yes	17	4.8
No	336	95.2
Using tamoxifen/raloxifene		
Yes	32	9.1
No	321	90.9

*BMI*: body mass index, *STI*: sexually transmitted infections, COCs: Combined Oral Contraceptive

### Comparison of HLB by sample characteristics

The median HLB score was 145 (Table 3). The median HLB score was significantly higher in women who worked as an assistant professor, considered the age of menarche and menopause as risk factors for women cancers, consumed regular snacks, received information about GCs, and had regular pap-smears (*p* < 0.005). In addition, there was a significant difference between median HLB sub-dimension scores and many variables such as working position, knowing risk factors for GCs, having regular examination and screening, regular nutrition, and exercise, etc. Table 4 includes all the statistical results related to HLB sub-dimensions and these variables.

**Table 3:** Distribution of the total and subscales of the healthy lifestyle behaviors scale (HLSBS) and gynecologic cancer awareness scale (GCCAS) of Female Academics

Scales and subscales	Median (Min–Max)
Healthy lifestyle behaviors scale	
Health responsibility	25 (10–36)
Physical activity	18 (8–32)
Nutrition	23 (13–34)
Spiritual development	28 (11–36)
Interpersonal relationship	29 (17–36)
Stress management	20 (8–32)
HLBS Total	145 (97–194)
Gynaecological Cancer Awareness Scale	
Routine control and serious disease perception awareness in GCs	99 (35–110)
GCs risk awareness	33 (12–45)
Awareness of prevention from GCs	22 (7–30)
Early diagnosis and information awareness in GCs	20 (8–20)
GCAS Total	172 (100–205)

**Table 4:** Evaluation of some characteristics of female academics and total and subscale scores of the healthy lifestyles behaviors scale

Characteristics	HR		PA		Nutr		SG		IR		SM		STOTAL	
	Median (Min–Maks)	*p*	Median (Min–Maks)	*p*	Median (Min–Maks)	*p*	Median (Min–Maks)	*p*	Median (Min–Maks)	*p*	Median (Min–Maks)	*p*	Median (Min–Maks)	*p*
Position														
Research assistant	25 (14–34)	**0.020**	17 (8–31)	**0.012**	23 (13–33)	**0.005**	27 (11–36)	**0.004**	29 (18–36)	0.305	19 (8–31)	0.165	141 (98–189)	**0.009**
Research assistant PhD	28 (18–32)		20 (10–30)		24 (18–30)		29 (23–35)		30 (25–35)		20 (9–28)		150 (117–178)	
Lecturer	25 (15–34)		23 (9–32)		22.5 (13–33)		28 (18–36)		29 (21–36)		21 (13–26)		149 (110–179)	
Assistant professor	27 (10–35)		18 (8–31)		25 (15–32)		30 (17–36)		30 (17–36)		21 (12–32)		152 (97–194)	
Associate professor	26 (15–34)		17 (8–28)		22.5 (13–31)		26 (17–36)		30 (18–36)		21 (11–27)		142 (97–181)	
Professor	24 (11–36)		18 (9–31)		24 (13–34)		29 (20–36)		29 (19–35)		20 (13–29)		146 (97–189)	
Smoking														
No	25 (10–36)	0.737	18 (8–32)	0.591	23 (13–34)	0.088	28 (11–36)	0.093	29 (17–36)	0.911	20 (8–32)	0.224	145 (97–194)	0.235
Yes	25 (14–33)		18 (8–28)		22 (13–32)		27 (18–36)		29 (18–36)		20 (11–30)		142.5 (104–182)	
Alcohol														
No	26 (11–35)	0.904	19 (8–32)	**0.033 **	23 (13–33)	0.929	29 (17–36)	0.076	29 (17–36)	0.092	20 (9–31)	0.272	145 (97–194)	0.413
Yes	25 (10–36)		17 (8–31)		23 (14–34)		28 (11–36)		30 (18–36)		21 (8–32)		146 (98–192)	
Chronic disease														
No	25 (10–35)	**0.040**	18 (8–32)	0.728	23 (13–33)	**0.036 **	28 (11–36)	0.707	29 (18–36)	0.894	20 (8–31)	0.499	145 (97–194)	0.230
Yes	26 (15–36)		19 (8–31)		24 (16–34)		28 (17–36)		29 (17–36)		21 (9–32)		146 (97–192)	
Regular exercises														
No	25 (11–35)	0.220	15 (8–29)	**< 0.001**	23 (13–32)	**0.020 **	28 (11–36)	**< 0.001 **	29 (17–36)	0.371	19 (8–31)	**< 0.001 **	139.5 (97–194)	**< 0.001**
Yes	26 (10–36)		24 (13–32)		24 (14–34)		30 (21–36)		30 (20–36)		21 (13–32)		153 (111–192)	
Regular main meal consumption														
No	25 (13–34)	**0.037 **	17 (8–31)	**0.028**	22 (13–32)	**< 0.001**	28 (11–36)	**0.009**	29 (17–36)	0.109	20 (8–31)	**0.004 **	142 (97–194)	**< 0.001**
Yes	26 (10–36)		19 (8–32)		25 (13–34)		29 (19–36)		30 (18–36)		21 (12–32)		150 (102–192)	
Regular snack meal consumption														
No	25 (10–34)	** < 0.001**	17 (8–31)	**< 0.001 **	22 (13–32)	**< 0.001 **	28 (11–36)	**< 0.001 **	29 (17–36)	** 0.011**	20 (8–31)	**< 0.001**	141 (97–194)	**< 0.001**
Yes	26.5 (16–36)		21 (9–32)		26 (18–34)		30.5 (20–36)		30 (18–36)		22 (13–32)		154.5 (114–192)	
Regular gynecological examination														
No	24 (10–34)	**< 0.001 **	17 (8–32)	0.305	23 (13–33)	**0.029**	28 (15–36)	0.638	29 (17–36)	0.278	20 (8–31)	0.883	143.5 (97–194)	**0.023 **
Yes	27 (15–36)		18 (8–31)		24 (14–34)		28 (11–36)		29 (18–36)		20 (9–32)		150 (98–192)	
Regular pap–smear examination														
No	24 (10–35)	** < 0.001**	18 (8–32)	0.359	23 (13–33)	0.118	28 (11–36)	0.772	29 (17–36)	0.192	21 (8–31)	0.725	144 (97–194)	**0.048**
Yes	27 (14–36)		18 (8–31)		24 (13–34)		29 (18–36)		30 (18–36)		20 (9–32)		149.5 (101–192)	
Information on gynecological cancers														
No	23 (11–33)	** < 0.001**	16 (8–29)	**< 0.001**	23 (13–32)	**0.035 **	27 (11–36)	** < 0.001**	29 (17–36)	**0.018 **	19 (12–30)	**< 0.001 **	138 (97–179)	**< 0.001 **
Yes	26 (10–36)		19 (8–32)		24 (13–34)		29 (15–36)		30 (18–36)		21 (8–32)		151 (101–194)	
Age of menarche is a risk factor for female cancers														
No	25 (10–34)	**0.00**1	17 (8–32)	** < 0.001**	23 (13–32)	** 0.005**	28 (11–36)	**0.012 **	29 (18–36)	0.508	20 (9–31)	0.153	142 (97–186)	**< 0.001 **
Yes	26 (11–36)		19 (8–31)		24 (13–34)		29 (15–36)		29 (17–36)		21 (8–32)		149 (97–194)	
Menstruation status														
No	24 (16–32)	** 0.004**	16 (8–29)	**0.003 **	22 (15–32)	**0.012**	28 (18–36)	0.288	29 (18–36)	0.152	20 (12–31)	0.171	139 (104–186)	**0.002 **
Yes	26 (10–36)		19 (8–32)		24 (13–34)		28 (11–36)		29 (17–36)		21 (8–32)		148 (97–194)	
Pregnancy experiencing														
No	25 (13–35)	** 0.008**	18 (8–32)	0.616	23 (13–33)	**0.002**	27.5 (11–36)	**0.003 **	29 (18–36)	0.247	20.5 (8–32)	0.582	144 (98–192)	0.070
Yes	26 (10–36)		18 (8–31)		24 (13–34)		29 (17–36)		30 (17–36)		20 (9–31)		149 (97–194)	
Infertility diagnosis														
No	25 (10–36)	**0.012 **	18 (8–32)	0.697	23 (13–34)	0.792	28 (11–36)	0.639	29 (18–36)	0.346	20 (8–32)	0.492	145 (97–194)	0.415
Yes	27 (19–34)		18 (9–25)		23 (18–31)		29 (17–34)		30 (17–36)		20 (12–24)		150 (97–174)	
STI diagnosis														
No	26 (10–36)	0.487	18 (8–32)	0.715	23.5 (13–34)	**0.027 **	28 (11–36)	0.067	29 (17–36)	0.488	20 (8–32)	0.274	145 (97–194)	0.137
Yes	25 (19–29)		19 (8–29)		22 (16–28)		25 (18–34)		28 (18–35)		18 (15–24)		136 (106–163)	

*HR* Health responsibility, *PA* Physical activity, *Nutr* Nutrition, *SD* Spiritual development, *IR* Interpersonal relations, *SM* Stress management, *STOTAL* Healthy lifestyles behaviors scale total

### Comparison of GCA by sample characteristics

The median GCA score was 172 (Table 5). The median GCA score was significantly higher in women who worked as an assistant professor, considered the age of menarche and menopause as risk factors for women cancers, received information about GCs, and experienced pregnancy process (p < 0.05). There was a significant difference between median GCA sub-dimension scores and many variables such as working position, knowing risk factors for GCs, having regular examination and screening, regular nutrition, and exercise, getting an infertility diagnosis, etc. Table 5 includes all the statistical results related to HLB sub-dimensions and these variables.

**Table 5:** Evaluation of gynecologic cancers awareness scale total and subscale scores with some characteristics of female academicians

Characteristics*	RCSDP		RA		AP		EDİA		GCATOTAL	
	Median (Min-Maks)	*p*	Median (Min-Maks)	*p*	Median (Min-Maks)	p	Median (Min-Maks)	p	Median (Min-Maks)	p
Position										
Research assistant	95 (35–110)	**0.001**	31.5 (13–45)	**0.012**	22 (9–30)	**< 0.001**	20 (10–20)	**0.020**	167 (104–202)	**< 0.001**
Research assistant. PhD	99 (78–110)		30 (20–45)		22 (15–29)		20 (16–20)		173 (144–201)	
Associate Professor	99.5 (67–110)		34 (15–45)		23 (13–28)		20 (12–20)		174 (125–197)	
Assistant professor	103 (62–110)		35 (12–45)		25 (7–30)		20 (12–20)		184 (130–205)	
Lecturer	98.5 (56–110)		32 (12–44)		22 (12–30)		20 (13–20)		167 (107–200)	
Professor	96 (54–110)		34 (17–45)		23 (13–30)		20 (8–20)		172 (100–202)	
Smoking										
No	98 (35–110)	0.399	33 (12–45)	**0.049**	23 (9–30)	**< 0.001**	20 (8–20)	0.158	173 (100–205)	0.073
Yes	100 (51–110)		30 (12–45)		20.5 (7–28)		20 (12–20)		165.5 (107–202)	
Alcohol										
No	97 (35–110)	0.401	34 (12–45)	**0.014**	23 (12–30)	**0.025**	20 (8–20)	0.722	173 (100–205)	0.186
Yes	100 (51–110)		31 (12–45)		22 (7–30)		20 (10–20)		168.5 (107–202)	
Regular snack meal consumption										
No	98 (35–110)	0.055	32 (12–45)	0.098	22 (9–30)	**0.007**	20 (8–20)	0.812	169 (100–205)	**0.021**
Yes	100 (55–110)		34 (15–45)		24 (7–30)		20 (12–20)		176 (116–202)	
Age is a risk factor for female cancers										
No	95 (51–110)	**< 0.001**	30 (12–45)	**< 0.001**	21.5 (9–30)	**0.001**	20 (8–20)	**0.001**	163.5 (100–205)	**< 0.001**
Yes	100 (35–110)		34 (13–45)		23 (7–30)		20 (14–20)		175 (104–204)	
Regular gynecological examination										
No	97 (35–110)	**0.001**	33 (12–45)	0.249	21 (7–30)	**< 0.001**	20 (10–20)	**0.030**	168 (104–203)	**0.002**
Yes	100 (54–110)		32 (12–45)		24 (14–30)		20 (8–20)		175 (100–205)	
Regular pap-smear examination										
No	97 (35–110	**< 0.001**	32 (12–45)	0.957	21 (7–30)	**< 0.001**	20 (10–20)	0.060	167 (104–203)	**< 0.001**
Yes	100.5 (54–110)		33 (12–45)		24 (14–30)		20 (8–20)		175 (100–205)	
Information on gynecological cancers										
No	95 (35–110)	**< 0.001**	31 (12–45)	**0.003**	21 (7–30)	**< 0.001**	20 (8–20)	**0.006**	164.5 (100–202)	**< 0.001**
Yes	100 (56–110)		34 (12–45)		24 (10–30)		20 (12–20)		176 (107–205)	
Age of menarche is a risk factor for female cancers										
No	95 (51–110)	** < 0.001**	29 (12–45)	**< 0.001**	21 (9–30)	** < 0.001**	20 (8–20)	**< 0.001**	162 (100–204)	**< 0.001**
Yes	102 (35–110)		36.5 (17–45)		24 (7–30)		20 (10–20)		182 (104–205)	
Age of menapouse is a risk factor for female cancers										
No	96.5 (54–110)	**0.007**	28 (12–45)	**< 0.001**	20.5 (13–30)	** < 0.001**	19.5 (8–20)	**< 0.001**	161.5 (100–202)	**< 0.001**
Yes	100 (35–110)		34 (12–45)		24 (7–30)		20 (12–20)		175 (104–205)	
Having polygamy in the past										
No	99 (35–110)	0.388	33 (12–45)	**0.002**	23 (9–30)	0.063	20 (8–20)	0.289	173 (100–205)	**0.023**
Yes	98.5 (55–110)		30 (16–45)		22 (7–28)		20 (12–20)		165 (116–198)	
Pregnancy experiencing										
No	95 (35–110)	**< 0.001**	31 (13–45)	**< 0.001**	21 (7–30)	**< 0.001**	20 (10–20)	**0.002**	165 (104–203)	**< 0.001**
Yes	102 (54–110)		34 (12–45)		24 (10–30)		20 (8–20)		177 (100–205)	
Infertility diagnosis										
No	99 (35–110)	0.089	33 (12–45)	0.108	22 (7–30)	0.053	20 (8–20)	**0.017**		**0.034**
Yes	104 (75–110)		34 (23–45)		26 (16–30		20 (18–20)		179 (156–202)	
Use COCs										
No	98 (35–110)	**0.024**	33 (13–45)	0.820	22 (9–30)	0.655	20 (8–20)	0.237	170 (100–203)	0.164
Yes	100 (58–110)		32 (12–45)		23 (7–30)		20 (12–20)		173 (123–205)	

*RCSP* Routine control and serious disease perception awareness in GCs

*RA* GC Risk Awareness

*AP* Awareness of prevention from GCs

*EDIA* Early diagnosis and information awareness in GCs

*GCA TOTAL* Gynecological Cancers Awareness Scale Total

### Comparison of demographic characteristics and HLB and GCA

The results of the correlation analysis between the demographic characteristics of the participants and the total scores and sub-dimension scores of the HLBs Scale and GCA Scale, are given in Table 6. According to the analysis, there was a weak correlation between the age and ‘Nutrition’ and ‘Spiritual Development’ sub-dimension scores, and there was a weak correlation between the height and ‘Interpersonal Relationships’, ‘Awareness of Routine Control and Serious Disease Perception in Gynecological Cancers’, ‘Awareness of Gynecological Cancer Risks’, ‘Awareness of Early Diagnosis and Information Awareness in Gynecological Cancers’ and the total score of the Gynecological Cancers Awareness Scale (*p* < 0.05). While no statistically significant relationship was observed in all scales in terms of weight variable, there was a weak negative relationship between BMI and only ‘Physical Activity’ sub-dimension (Table 7).

**Table 6:** The relationship between demographic characteristics of participants and healthy lifestyles behaviours scale and gynecological cancers awareness scale and subscales

Scales	Age	Height	Weight	BKI
Health responsibility				
r	0.036	0.047	0.002	– 0.019
p	0.498	0.382	0.972	0.725
Physical activity				
r	0.041	0.098	– 0.091	**− 0.130**
p	0.446	0.065	0.088	**0.014**
Nutrition				
r	0.134	0.017	– 0.051	– 0.059
p	**0.012**	0.753	0.340	0.269
Spiritual Development				
r	0.105	0.032	0.022	0.010
p	**0.049**	0.548	0.686	0.850
Interpersonal relationship				
r	– 0.010	0.125	0.013	– 0.040
p	0.853	**0.019**	0.801	0.451
Stress management				
r	0.087	0.046	– 0.054	– 0.075
p	0.103	0.388	0.312	0.161
Healthy lifestyle behaviours scale total				
r	0.088	0.085	– 0.040	– 0.076
p	0.097	0.112	0.449	0.154
Routine control and serious disease perception awareness in GCs				
r	0.015	0.120	0.050	– 0.008
p	0.776	**0.024**	0.354	0.882
Risk Awareness in GCs				
r	0.033	0.132	0.069	0.012
p	0.534	**0.013**	0.195	0.825
Awareness of prevention from GCs				
r	0.045	0.098	0.023	– 0.021
p	0.404	0.066	0.668	0.699
Early diagnosis and information awareness in GCs				
r	– 0.010	0.107	0.031	– 0.017
p	0.856	**0.045**	0.568	0.750
Gynecologic cancers awareness scale total				
r	0.031	0.155	0.064	– 0.007
p	0.563	**0.003**	0.231	0.890

**Table 7:** The relationship between healthy lifestyles behaviours and subscales of female academics and gynecologic cancers awareness scale and subscales

Scales		1	2	3	4	5	6	7	8	9	10	11	12
1-HR	r p	1	0.352 ** < 0.001**	0.394 ** < 0.001**	0.440 ** < 0.001**	0.513 ** < 0.001**	0.413 ** < 0.001**	0.710 ** < 0.001**	0.396 ** < 0.001**	0.204 ** < 0.001**	0.468 ** < 0.001**	0.146 **0.006**	0.449 ** < 0.001**
2-PA	r p		1	0.374 ** < 0.001**	0.378 ** < 0.001**	0.270 ** < 0.001**	0.571 ** < 0.001**	0.708 ** < 0.001**	0.068 0.201	0.138 **0.010**	0.225 ** < 0.001**	0.101 0.058	0.154 **0.004**
3-Nutr	r p			1	0.401 ** < 0.001**	0.354 ** < 0.001**	0.443 ** < 0.001**	0.667 ** < 0.001**	0.215 ** < 0.001**	0.159 **0.003**	0.331 ** < 0.001**	0.117 **0.028**	0.282 ** < 0.001**
4-SG	r p				1	0.643 ** < 0.001**	0.601 ** < 0.001**	0.775 ** < 0.001**	0.147 **0.006**	0.134 **0.012**	0.349 ** < 0.001**	0.052 0.330	0.228 ** < 0.001**
5-IR	r p					1	0.502 ** < 0.001**	0.724 ** < 0.001**	0.237 ** < 0.001**	0.093 0.082	0.251 ** < 0.001**	0.086 0.108	0.251 ** < 0.001**
6-SY	r p						1	0.802 ** < 0.001**	0.181 **0.001**	0.126 **0.018**	0.283 ** < 0.001**	0.022 0.679	0.229 ** < 0.001**
7-STOTAL	r p							1	0.278 ** < 0.001**	0.196 ** < 0.001**	0.434 ** < 0.001**	0.122 **0.022**	0.360 ** < 0.001**
8-RCSP	r p								1	0.271 ** < 0.001**	0.500 ** < 0.001**	0.369 ** < 0.001**	0.888 ** < 0.001**
9-RA	r p									1	0.339 ** < 0.001**	0.342 ** < 0.001**	0.631 ** < 0.001**
10-AP	r p										1	0.242 ** < 0.001**	0.697 ** < 0.001**
11-EDİA	r p											1	0.504 ** < 0.001**
12-GCATOTAL	r p												1

*HR*: Health Responsibilty, *PA*: Physical Activity, Nutr: Nutrition, *SG*: Spiritual Development, *IR*: Interpersonal Relationship, *SM*: Stress Management, *HLBS* Total: Healthy Lifestyle Behaviors Scale Total, *RCSP*: Routine Control and Serious Disease Perception Awareness in GCs, *RA:GC* Risk Awareness in GCs, *AP*: Awareness of Prevention from GCs, EDİA: Early Diagnosis and Information Awareness in GCs.,GCA TOTAL: Gynecologic Cancers Awareness Scale Total

### Correlation between HLB and GCA

The median HLB score was positively and moderately correlated with the GCA (p < 0.05). Other data on the correlation of the sub-dimensions of both scales with each other are given in Table 6.

## Discussion

The number of women affected by GCs is increasing day by day. Therefore, the effect of women’s knowledge on GC risk factors and symptoms in creating HLBs to prevent cancer has a significant importance [41]. Considering the available literature, although GCA has been investigated in women with gynecological problems [42] or in the general population [14, 15], there are no studies evaluating both together and the relationship between them in women. To the best of our knowledge, this is the first study examining the HLBs and GCA levels of women academicians and investigating the relationship between them.

In the current study, women’s HLBs level was close to moderate, and GCA level was high, and both were affected by some characteristics (such as working position, exercising regularly, consuming regular meals and snacks, regular gynecological examination, and pap-smear, getting information about GCs and knowing risk factors, etc.). In addition, in the present study, as women’s HLBs levels increased, their GCA levels also increased.

The Council of Higher Education reported that as of 2021, the number of women academicians in Turkey is 83,860 and the rate of them is 46% [43]. They are an important population that can have a critical impact on the society due to their position, knowledge, skills, behavior, and higher education level. In this respect, they have a high status among all women in the society and play a key role in the development and maintenance of HLBs in the lives of their students and other women in the society, as well as in raising awareness of GCs.

In the current study, HLBs of women academicians were close to moderate level. Contrary to this finding, Göger and Cingil (2022) [44] reported that HLBs of women aged 18–49 were low level. Similarly, in the study conducted by Gözüyeşil et al. (2020) [45] with 425 women aged 15–49, they found that HLBs of women were low level. Considering the results of these studies, the difference between HLBs scores may be associated with the fact that the women in the present study had a higher education level and easier access to health services compared to other women in the society. Duman et al. (2013) [34] reported that the health motivation of women academicians is quite high. Previous studies found that GCA increased as women’s age and education level increased [14, 16, 39, 46]. Similarly, in our study, women academicians who had the highest educational level among women in the society had a high level of GCA. Doğan and Fışkın (2022) [37] found that women academicians’ perceptions of early diagnosis of cervical cancer and the usefulness of screening increased as the age increased. On the contrary, the GCA scores of women academicians working in assistant professor positions, representing the younger age group, were significantly higher in our study. This difference may be explained by the fact that the sample groups of these studies were different from each other. For example, in the study of Teskereci et al. (2022) [14], there were women who were aged between 20 and 65 years and were at least primary school graduates. Another example, Doğan and Fışkın (2022) [37] only included women academicians of one university. Whereas our study included women academicians from all universities in Turkey’s capital. Therefore, the fact that our sample group included a larger population, and a different group of academicians may have caused this difference. In this case, young academicians who have completed long and tiring process of postgraduate education, may have spent more time on their own health and this may have caused this difference. In addition, the higher scores of those who were the assistant professors may be since they represent a younger age group in higher education institutions, rapid access to information about health, and use digital health resources more effectively and the fact that cancer screening programs are accessible at a younger age in Turkey.

Adoption and lifelong implementation of HLBs (such as balanced and healthy diet, consuming regular meals and snacks, exercising regularly, having regular gynecological examinations) contribute to prevention and early diagnosis of chronic diseases, increase in life expectancy, prevention from cancer, and increase in quality of life [47–49]. HLBs such as consuming regular meals and snacks and exercising regularly help control the factors associated with nutrition and inactivity that pose a risk for cancer by providing appetite control, regulation of body weight, and blood glucose control [49]. It is one of the expected behaviors that individuals with high awareness of cancer should regulate their habits related to nutrition and exercise. Similarly, this study also confirmed that women had integrated such HLB and screenings into their lives, and that they took on their own health responsibilities.

Raising awareness about cancer, improving public awareness and rapid access to cancer screenings are among the most effective methods in the fight against cancer [50]. Furthermore, HLB are closely related to individuals’ knowledge, attitudes, and practices regarding health. Tang et al. (2022) [51] states that “knowledge” indicates the understanding and explanation of relevant knowledge, “attitudes” means appropriate beliefs and positive attitudes, and “practice” is behaviors that change to achieve a health goal. So, it can be said that when people have sufficient and reliable information and have a strong sense of desire, it may be possible to adopt and maintain HLB. Driessenet al. (2022) [52] found that women experienced with GCs especially adopted HLBs in their lives. Keaver et al. (2021) [53] reported that about 84.4% of the breast cancer survivors exhibited at least 1 positive behavior for improving nutrition and physical activity after cancer diagnosis or treatment. Moreover, another important point that increases the GCA is to have information about GC [14]. In parallel with this finding, women who received information about GC had higher GCA and HLB scores. Therefore, it can be said that women with high GCA have adopted and maintained HLB to prevent GCs.

Advanced age, age at menarche and/or menopause are non-modifiable risk factors for GC [54]. In our study, women who expressed these risk factors as risk factors for GC had higher GCA scores. We also found that women academicians with a history of infertility, pregnancy, multiple sex partners, who had regular gynecological examination and regular pap-smear test had higher GCA scores. These findings in our study were similar to previous studies. Özcan and Doğan (2021) [12] highlighted that as women’s ages for marriage, first sexual intercourse and first pregnancy increased, their GCA levels increased. Moodley et al. (2020) [55] found that women who did not have or have a sexual partner had a significantly lower awareness of cervical cancer symptoms. In another study, the majority (54.8%) of the participants had between 0 and 1 sexual partner, while those with more than one partner were about five times more likely to turn up for cervical cancer screening [56]. Doğan and Fışkın (2022) [37] indicated that women academicians were more susceptible to attitude towards early diagnosis of cervical cancer as their education level increased. In another study, it was indicated that women with a master’s degree and higher had higher perception of benefit and motivation and lower susceptibility and pap smear barrier perception scores [57]. Consistent with previous studies [45, 58, 59], we believe that higher education level influences the fact that these risk factors are known by women with high GCA scores.

Finally, we found that as women’s HLB levels increased, their GCA levels also increased. Similarly, Başoğlu (2021) [59] found that the as GCA level of lesbian and bisexual women increased, their HLB increased. Undoubtedly, education and income levels have a significant effect on people’ taking their own health responsibilities in matters related to preventing diseases and promoting health [61, 62]. However, in general, it is thought that raising awareness of all women in the society about early diagnosis methods, symptoms and risk factors, and the importance of routine control for GC may be contributed to adopt HLB in their lives.

## Implications for practice and policy

Based on all these results, it is recommended that comprehensive and systematically planned health trainings be organized by nurses working in the field of women’s health to participate in gynecological cancer screening programs and to increase behaviors for early diagnosis and prevention. In cooperation with health institutions and screening programs, awareness can be raised by providing easy access to screening services to women academicians through mobile screening units that regularly visit universities. Health policy makers can make GC screening programs more accessible, considering the needs of women academicians. Short videos and posters can be shared on digital platforms and social media within the university. Social support groups for female academics can be established to encourage healthy living behaviors and ensure sustainability. Academicians with high level of awareness can be encouraged to mentor other female employees. Annual health programs including seminars, workshops, exercise activities and healthy nutrition guides can be designed for women academicians. At the end of the program, the impact of the program can be evaluated by following the behavioral changes of the participants. Academicians with high level of awareness can be encouraged to mentor other women academicians. In addition, ‘Women’s Health and Early Diagnosis’ in academic settings compulsory education programs and awareness-raising campaigns on the subject can be promoted. In cooperation with the Ministry of Health, universities and civil society organizations, arrangements can be made to facilitate access to health services for women academicians. For example, days off and other facilities could be provided to encourage participation in screening programs. Finally, the establishment of national databases on women’s health and the use of these data in policy development will contribute to the planning of more effective health strategies. Thus, such comprehensive and systematic approaches will enable not only women academicians but also other women in the society to adopt HLBs and increase GCA.

## Limitations

This study had two limitations. First, although there were women academicians who were working at a university in Ankara, since the study was an online study, it was not known how many people the questionnaires reached in total, but a total of 353women academicians answered. In addition, although the data do not reflect the whole of Turkey, they do reflect the vast majority. This and the fact that the current study conducted by the online survey, implied that findings cannot be generalized of all the cities in Turkey, HLB or GCA. Further research should be carried out to access a wider range of women academicians working across cities and even different countries. Second, due to the nature of descriptive and correlational studies, the actual causal effects could not be interpreted regarding HLB and GCA.

## Conclusion

The findings of this study highlight the importance of promoting healthy lifestyle behaviors and increasing awareness of gynecological cancers, particularly among women academicians. We conclude that high HLB levels are associated with high GCA in women academicians. In addition, working position, exercising regularly, consuming regular meals and snacks, regular gynecological examination, and pap-smear, getting information about GCs and knowing risk factors created significant differences on both HLB and GCA levels. In addition, further studies could focus on larger and more diverse populations to increase the generalizability of these findings and explore tailored interventions that address the unique needs of women in various demographic and other occupational groups. Such efforts can contribute to the development of more inclusive health promotion strategies and policies at a societal level.

## Data Availability

The data that support the findings of this study are not openly available due to reasons of sensitivity and are available from the corresponding author upon reasonable request.
